# Glycolysis-dependent reactive oxygen species mediate desmopressin acetate–induced rescue of platelet dysfunction caused by antiplatelet therapy

**DOI:** 10.4103/mgr.MEDGASRES-D-25-00353

**Published:** 2026-03-26

**Authors:** Kaiwen Wang, Zheng Wen, Shaohua Mo, Kaige Zheng, Jun Wu, Yang Liu, Jiaming Zhang, Yi Yang, Yanan Zhang, Shuo Wang, Qingyuan Liu, Xianzeng Tong

**Affiliations:** 1 Department of Neurosurgery, Beijing Friendship Hospital, Capital Medical University, Beijing, China; 2 Department of Neurosurgery, Beijing Tiantan Hospital, Capital Medical University, Beijing, China; 3 China National Clinical Research Center for Neurological Diseases, Beijing, China; 4 Department of Blood Transfusion, Beijing Tiantan Hospital, Capital Medical University, Beijing, China

**Keywords:** coagulation impairment, desmopressin, dual antiplatelet therapy, glycolysis, metabolomics, oxidative stress, peroxiredoxin-5, platelet dysfunction, proteomics, reactive oxygen species

## Abstract

Antiplatelet therapy is extensively used in the prevention and treatment of cardiovascular and cerebrovascular diseases; however, life-threatening hemorrhage requires urgent reversal of platelet dysfunction. Desmopressin acetate has been proposed as a rescue strategy, yet its efficacy and underlying mechanisms remain incompletely understood, particularly regarding redox regulation. A mouse carotid artery blood flow injury model was employed to evaluate the effects of desmopressin acetate on platelet and coagulation dysfunction induced by antiplatelet therapy. Proteomic analyses were performed in both patients and mice to identify differentially expressed proteins. Genetic knockout and pharmacological inhibition approaches were used to investigate the mechanistic pathways involved. Desmopressin acetate effectively restored platelet function and coagulation capacity in antiplatelet-treated mice. Proteomic profiling identified peroxiredoxin-5, a key antioxidant enzyme, as significantly upregulated following antiplatelet therapy but markedly downregulated after desmopressin acetate administration; these findings were validated in plasma samples from 10 patients who received dual antiplatelet therapy for unruptured intracranial aneurysms. Functional studies demonstrated that proteomic profiling identified peroxiredoxin-5 supplementation impaired platelet function, whereas proteomic profiling identified peroxiredoxin-5 knockout or inhibition significantly improved platelet activity. Notably, desmopressin acetate primarily suppressed liver-derived proteomic profiling identified peroxiredoxin-5 expression. Mechanistically, desmopressin acetate enhanced platelet glycolysis via phosphofructokinase-2/fructose-2,6-bisphosphatase 3 activation, leading to increased intracellular reactive oxygen species levels. Inhibition of phosphofructokinase-2/fructose-2,6-bisphosphatase 3 attenuated glycolysis, reduced reactive oxygen species generation, and restored proteomic profiling identified peroxiredoxin-5 expression, thereby abolishing the platelet-rescuing effects of desmopressin acetate. Desmopressin acetate rescued platelet dysfunction induced by antiplatelet therapy through a glycolysis–reactive oxygen species–proteomic profiling identified peroxiredoxin-5 axis, in which glycolysis-driven reactive oxygen species generation plays a central regulatory role. These findings indicate redox modulation as a critical mechanism underlying desmopressin acetate-mediated platelet rescue and suggest a potential therapeutic strategy for managing severe bleeding associated with antiplatelet therapy.

## Graphical abstract

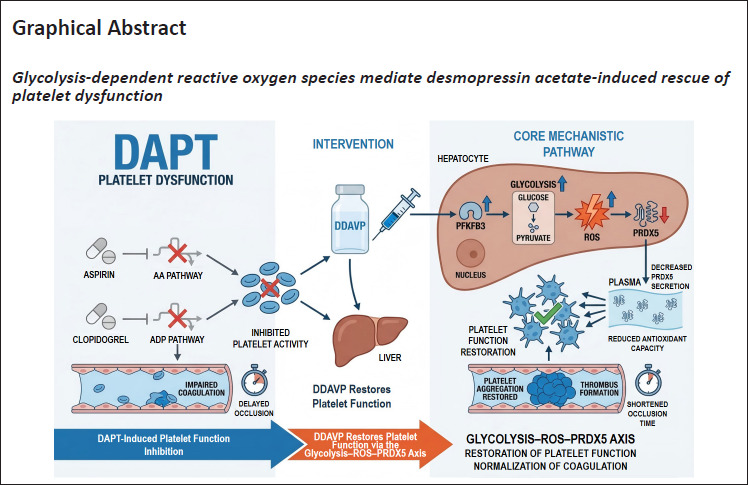

## Introduction

Antiplatelet therapy is commonly employed for the prevention and treatment of cardiovascular and cerebrovascular diseases by inhibiting platelet function.^1^,^2^ However, in conditions requiring surgery, such as severe intracerebral hemorrhage and traumatic brain injury, coagulation disorders resulting from platelet dysfunction related to antiplatelet therapy may lead to challenging intraoperative hemostasis and bleeding, as well as postoperative rebleeding. Previous research has indicated that a preoperative history of antiplatelet therapy, particularly dual antiplatelet therapy (DAPT), may significantly increase the risk of postoperative rebleeding.^3^ Additionally, there exists a causal link between platelet dysfunction and postoperative rebleeding.^4^ Therefore, there is an urgent clinical need to address platelet dysfunction induced by antiplatelet therapy promptly.

Platelet dysfunction largely reflects impaired platelet activation secondary to pharmacological blockade of key signaling pathways. Platelet activation primarily involves the arachidonic acid (AA) pathway, adenosine diphosphate (ADP) pathway, and glycoprotein receptor pathway.^5^ Commonly used antiplatelet medications include aspirin (targeting the AA pathway), clopidogrel (targeting the ADP pathway), and others, which inhibit platelet activation and thereby contribute to platelet dysfunction. Previous clinical studies have demonstrated that desmopressin acetate (DDAVP) can ameliorate antiplatelet-associated platelet dysfunction and prevent hematoma expansion in patients with intracerebral hemorrhage who are receiving antiplatelet therapy.^6^,^7^,^8^ DDAVP is known to enhance hemostasis by promoting von Willebrand factor release and facilitating platelet adhesion and activation.^9^ However, limited research has explored the underlying mechanism through which DDAVP improves platelet dysfunction associated with antiplatelet therapy.

Oxidative stress is integral in platelet activation and can also induce platelet pre-activation.^10^ It gives rise to the generation of reactive oxygen species (ROS), which can directly or indirectly trigger platelet activation.^11^ ROS can act as secondary messengers in the process of platelet activation.^12^,^13^ Excessive production of ROS can lead to hyperactivation of platelets and formation of blood clots.^13^,^14^,^15^ We hypothesize that DDAVP may alleviate platelet dysfunction by modulating systemic oxidative stress.

The peroxiredoxin (PRDX) family comprises antioxidant enzymes that regulate intracellular redox balance in response to oxidative stress.^16^,^17^,^18^ Among them, PRDX5 effectively scavenges ROS and thereby modulates platelet activation, suggesting a potential role in antiplatelet-associated platelet dysfunction.^19^,^20^

In this study, utilizing a mouse model of carotid artery blood flow and a clinical cohort, the research aims to investigate whether DDAVP could improve the platelet dysfunction caused by antiplatelet therapy. Through the integration of proteomics and metabolomics analysis, the pathways associated with the rescue effect of DDAVP for antiplatelet therapy will be thoroughly examined.

## Methods

### Study design

To validate whether DDAVP could rescue the platelet and coagulation dysfunction caused by antiplatelet therapy and investigate its underlying mechanism deeply, this study performed the following analyses: (1) comparison of platelet and coagulation function before and after the use of DDAVP based on the mouse model of DAPT; (2) investigation of proteins related to platelet and coagulation function rescue by DDAVP built on the mouse model of DAPT and a clinical cohort of patients receiving DAPT; and (3) analysis of the pathway of DDAVP to rescue the platelet and coagulation dysfunction caused by DAPT with proteomic and metabonomic methods. This study has been reported in accordance with the Animals in Research: Reporting *In Vivo* Experiments (ARRIVE) guidelines.^21^

### Clinical cohort and blood sample collection

Approval was granted by the Institutional Review Board of Tiantan Hospital (approval No. KY2022-226; February 6, 2023). Informed consent was obtained from all individual participants included in the study.

The discovery cohort prospectively enrolled 10 patients at Beijing Tiantan Hospital who were prepared to receive stent-assisted coiling for unruptured intracranial aneurysms from November 2021 to December 2021 from the 100-Project phase-I cohort (unique identifier: NCT04872842), and therefore, they needed to receive DAPT. The inclusion and exclusion criteria were listed in **Additional file 1**. Patients in the discovery cohort received the standard DAPT (aspirin 100 mg once daily plus clopidogrel 75 mg once daily, for 7 days). Before DAPT and on 7 days after DAPT, blood samples were collected.

The derivation cohort also prospectively included 15 patients receiving DAPT because of planned stent-assisted coiling for unruptured intracranial aneurysms from January 2022 to August 2022 from the 100-Project phase-II cohort (unique identifier: NCT05608122). The inclusion and exclusion criteria, as well as blood sample collection, were the same as the discovery cohort.

The validation cohort recruited 372 patients who received standard DAPT currently from the 100-Project phase-II cohort (unique identifier: NCT05608122). The inclusion and exclusion criteria were detailed in **Additional file 1**. After final enrollment, these patients would receive a subcutaneous injection of 0.4 μg/kg DDAVP. Blood samples were then collected before DDAVP administration and at the 1^st^ hour and 6^th^ hour after DDAVP administration.

To ensure consistency, all patients were required to fast for 8 hours before blood sampling. After excluding blood samples with hemolysis, the remainder was centrifuged at 1500 × *g* for 10 minutes within 3 hours after collection. Any unused samples were quickly frozen in liquid nitrogen for 15 minutes and then transferred to a –80°C storage environment.

### Mouse models of dual antiplatelet therapy and desmopressin acetate treatment

Female C57BL/6J mice, aged 8 weeks and weighing between 18 and 22 g, were used to minimize variability related to sex-specific differences in platelet function and coagulation responses and purchased from Gempharmatech Co., Ltd. (Nanjing, China; approval No. SCXK (Su) 2023-0009). Animal experiments were approved by the Animal Ethics Committee of Beijing Institute of Neurosurgery, Capital Medical University (approval No. 202103005; March 6, 2023). The details of mouse models of DAPT and DDAVP treatment were described in **Additional file 1**. A mouse model of DAPT was established by the gavage of aspirin and clopidogrel. A mouse model of DDAVP therapy was established based on the mouse model of DAPT.^22^,^23^ After an appropriate DAPT, mice would receive an intraperitoneal injection of 3.6 μg/kg DDAVP. One hour later, these mice were used for further analysis.

Liver, kidney, and lung samples were obtained after deep anesthesia with isoflurane (induction: 4–5% (v/v); maintenance: 1.5–2.0% (v/v) in oxygen at 1.0–1.5 L/min, RWD Life Science Co., Ltd., Shenzhen, China) followed by cervical dislocation. All visible blood clots were removed, and tissue samples were washed with low-temperature phosphate-buffered saline solution to clean any residual blood cells. After this preparation, the samples were snap frozen and stored in liquid nitrogen. For blood collection, the retro-orbital bleeding method was used, and the collected samples were quickly subjected to further analysis.

### Mouse model of carotid artery blood flow

A mouse model of carotid artery blood flow was established by using ferric chloride (FeCl_3_)-induced injury model.^24^ The details of this model were provided in **Additional file 1**. Laser speckle contrast imaging (RFLS III, Full-Field Laser Perfusion Imager, RWD Life Sciences, Shenzhen, China) was employed to evaluate the blood flow of carotid artery before and after FeCl_3_ administration.^25^

### Knockout of PRDX5 and intervention by PRDX5

PRDX5 knockout (PRDX5^KO^, C57BL/6JGpt-Prdx5^em1Cflox^) mice (Strain No. T029084) were purchased from GemPharmatech Co., Ltd. Sixty PRDX5^KO^ female mice were used in experiments at 8 weeks of age and age-matched littermates or purchased C57BL/6J mice were used as wild type controls.

To assess the functional contribution of PRDX5, 12 WT mice and 12 PRDX5^KO^ mice were randomly assigned to the following groups: (1) WT + vehicle (*n* = 6), (2) WT + recombinant PRDX5 (*n* = 6), (3) PRDX5^KO^ + vehicle (*n* = 6), and (4) PRDX5^KO^ + recombinant PRDX5 (*n* = 6). Recombinant PRDX5 (HY-P73375, MCE, Shanghai, China) was administered intraperitoneally at 0.5 mg/kg, and the vehicle group received an equal volume of solvent (deionized water).

The biological effect of PRDX5 was pharmacologically inhibited by using a specific inhibitor (coenzyme A). One hour after PRDX5 administration, 5 mg/kg coenzyme A (HY-128851, MCE, Shanghai, China) in dimethyl sulfoxide or an equal volume of dimethyl sulfoxide was injected intraperitoneally. Subsequently, 1 hour after coenzyme A or dimethyl sulfoxide intervention, platelet and coagulation function were analyzed.

### Platelet morphology analysis

Platelets isolated from blood collected from WT, DAPT-treated, and DDAVP-treated mice was analyzed using transmission electron microscopy. The details were given in **Additional file 1**. Open canalicular system (OCS) and α-granules were observed and compared among different treatments.^26^

### Platelet function evaluation

For the clinical cohort, platelet function was measured by using the thromboelastogram (Rotem, TEM Innovations GmbH, Munich, Germany). The AA (AA channel) represented the inhibition of platelets by aspirin, and ADP (adenosine channel) represented the inhibition of platelets by clopidogrel.

For mice, platelet function was measured using platelet aggregation capability, which was assessed by light transmission aggregometry,^27^,^28^ and further detailed in **Additional file 1**.

### Cell culture, immunofluorescence staining and western blot assay

Liver cells (LO2 cells, RRID:CVCL_6926) were purchased from The Cell Bank of Type Culture Collection of Chinese Academy of Sciences, and were then cultured with Dulbecco’s modified Eagle medium (Thermo Fisher Scientific, Inc., Waltham, MA, USA) containing 10% fetal bovine serum (Gibco, Thermo, Sydney, NSW, Australia) and maintained at 37°C, 5% CO_2_, and saturated humidity. To investigate the biological effect of DDAVP on liver cells, LO2 cells were treated with 1.0 ng/kg DDAVP in deionized water or an equal volume of deionized water for 6 hours.

The details of immunofluorescence staining and Western blot were given in **Additional file 1**.

### Enzyme-linked immunosorbent assay

The culture supernatant of LO2 cells was collected for enzyme-linked immunosorbent assay. PRDX5 levels were quantified using a human PRDX5 enzyme-linked immunosorbent assay kit (Cat# xk-sjh793, Xkbio, Shanghai, China) according to the manufacturer’s instructions.

### Proteomics and metabolomic analysis

Proteomic analysis was performed on mouse plasma, human plasma, and LO2 cell homogenates, whereas metabolomic analysis was conducted on mouse plasma and LO2 cell homogenates. Fold change (FC) was calculated based on the signal intensity of proteins and metabolites.

As for proteomics analysis, proteins with a |log1.5(FC)| > 1, variable importance in projection (VIP) > 1, and *P* < 0.05 between two groups were considered significant. The ClueGo plug-in in CytoScape (version 3.6.1; https://cytoscape.org)^29^ was employed to perform functional enrichment analysis of altered proteins. Enrichment analysis was carried out using the Kyoto Encyclopaedia of Genes and Genomes database, while biological processes and molecular functions were analyzed using the Gene Ontology database.

Metabonomic analysis revealed that the metabolites with a |log2(FC)| >1, VIP > 1, and *P* < 0.05 between two groups were significant. The Analysis Module in MetaboAnalyst (https://www.metaboanalyst.ca/) was used for metabolite classification and pathway enrichment analysis.

### Statistical analysis

Statistical analyses were performed using SPSS (version 24.0, IBM Corp., Armonk, NY, USA) and GraphPad Prism (version 8.4.3, GraphPad Software, Boston, MA, USA, https:/www.graphpad.com). All tests were two-sided, and a *P* value < 0.05 was considered statistically significant.

Normality of continuous variables was assessed using the Shapiro–Wilk test prior to analysis. Normally distributed data are presented as mean ± standard deviation, whereas non-normally distributed data are presented as median with interquartile range. Categorical variables are reported as numbers and percentages.

For comparisons between two paired groups (e.g., before and after DAPT use), paired two-tailed Student’s *t*-tests were applied for normally distributed data, and Wilcoxon signed-rank tests were used otherwise. For comparisons between two independent groups, unpaired Student’s *t*-tests were used for normally distributed data, and Wilcoxon rank-sum tests were applied for non-normally distributed data.

Comparisons among three or more independent groups were performed using one-way analysis of variance followed by Tukey’s *post hoc* test for normally distributed data, or the Kruskal–Wallis test followed by Dunn’s multiple comparisons test for non-normally distributed data.

For repeated measurements across multiple time points, repeated-measures analysis of variance followed by Tukey’s *post hoc* test was used for normally distributed data, whereas the Friedman test followed by Dunn’s multiple comparisons test was applied for non-normally distributed data.

Receiver operating characteristic curve analysis was performed to evaluate diagnostic performance, and the area under the curve was calculated. Pairwise comparisons of the areas under the curve were conducted using DeLong’s test for correlated receiver operating characteristic curves. Exact *P* values were indicated in the corresponding figures where applicable.

## Results

### Desmopressin acetate rescues the platelet and coagulation dysfunction caused by dual antiplatelet therapy

**Figure 1:**
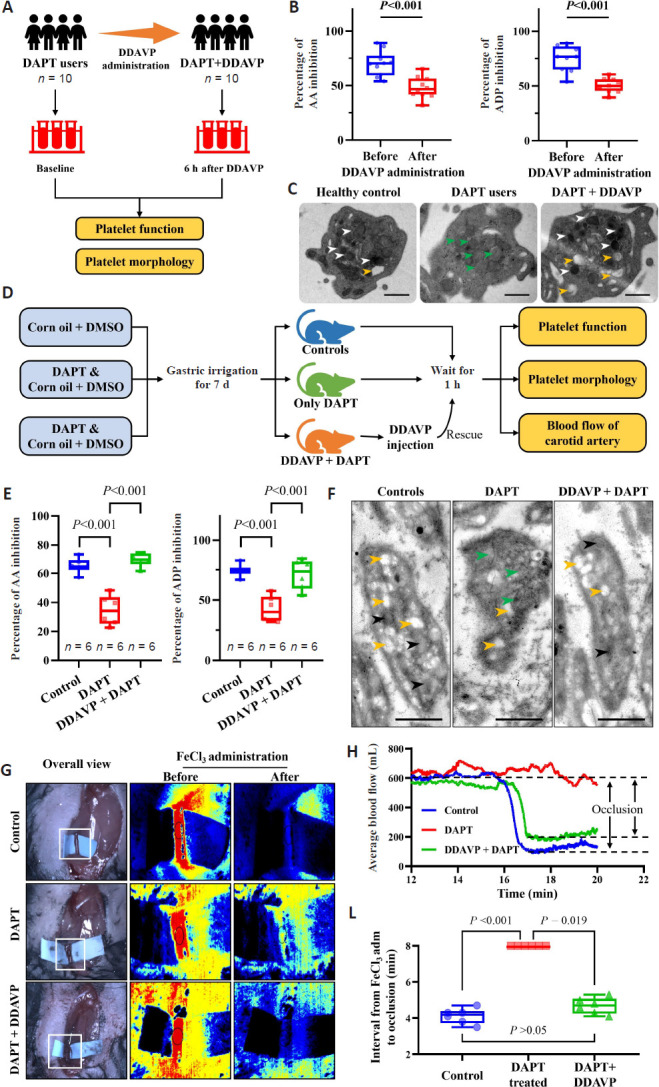
DDAVP rescues the platelet and coagulation dysfunction caused by DAPT. (A) The diagram illustrates the self-controlled study (discovery cohort) conducted to validate the changes in platelet function before and after DDAVP (0.4 μg/kg) treatment in patients on DAPT. (B) The platelet function examination before and after DDAVP treatment (6 hours later). It was observed that DDAVP could rescue the dysfunctional platelets caused by DAPT. Wilcoxon signed-rank test was used for within-subject comparisons. (C) The TEM images of the platelet morphology of the representative case in each group. Closed OCSs and fewer α granules were observed in the platelets of patients. After being treated by DDAVP, platelets showed increasing normal OCSs and more α granules. Yellow arrows indicate normal OCSs, green arrows indicate closed OCSs (non-functional), and white arrows indicate α granules Scale bars: 500 nm. (D) The diagram presents an *in vivo* study conducted to investigate the efficacy of DDAVP in rescuing platelet and coagulation dysfunction caused by antiplatelet therapy. The mouse model of carotid artery blood flow was utilized to evaluate the coagulation function. (E) The platelet function examinations, where AA inhibition indicates the inhibition caused by aspirin, and ADP inhibition indicates the inhibition caused by clopidogrel. Unpaired two-tailed Student’s *t*-test (or Wilcoxon rank-sum test when appropriate) was used for the indicated pairwise comparisons. (F) The TEM images present the platelet morphology of a representative case in each group. The platelets of mice treated by DAPT showed an increase in closed OCSs (non-functional) and a decrease in α granules, as compared to the control group. After being treated with DDAVP, the platelets showed an increase in normal OCSs (functional) and more α granules. Yellow arrows indicate normal OCSs, green arrows indicate closed OCSs (non-functional), and black arrows indicate α granules. Scale bars: 500 nm. (G) The images of laser speckle contrast imaging analysis of carotid artery blood flow in the control group (*n* = 4), DAPT group (*n* = 4), and DAPT + DDAVP group (*n* = 4). Over an observational period of 20 minutes, the laser speckle contrast imaging analysis revealed that DAPT can prevent the formation of clots in the artery, and DDAVP can rescue the coagulation dysfunction caused by DAPT. White boxes indicate ROIs used for quantitative analysis. Each circle represents one individual mouse. (H) The line plot presents the carotid artery blood flow after FeCl_3_ administration. No occlusion was observed in the mice of the DAPT group (*n* = 4), as compared to the mice of the control group (*n* = 4). However, after DDAVP treatment (*n* = 4), occlusion reoccurred in the mice receiving DAPT. (I) The boxplot presents the time interval from FeCl_3_ administration to occlusion. Starting at 12 minutes after FeCl_3_ usage, DDAVP treatment significantly rescues the no occlusion condition as compared to mice receiving DAPT. Statistical differences among groups were analyzed using the Kruskal–Wallis test with Dunn’s *post hoc* test. AA: Arachidonic acid; ADP: adenosine diphosphate; DAPT: dual antiplatelet therapy (aspirin plus clopidogrel); DDAVP: desmopressin acetate; DMSO: dimethyl sulfoxide; FeCl_3_: ferric chloride; OCS: open canalicular system; ROIs: regions of interests; TEM: transmission electron microscopic.

To assess whether DDAVP can alleviate the platelet and coagulation dysfunction induced by antiplatelet therapy, this research investigated the alteration in platelet function before and after DDAVP treatment in a discovery cohort comprising 10 patients receiving DAPT (**Figure 1A**). The platelet function examination confirmed the restoration of platelet function following DDAVP treatment in patients on DAPT (**Figure 1B**). Platelet morphology analysis demonstrated that DDAVP was able to rescue nonfunctional platelets (closed OCSs) and decrease α granule depletion caused by DAPT (**Figure 1C**). Details regarding the patients in the discovery cohort were provided in **Table 1**. Subsequently, the study established mouse models of DAPT (**Figure 1D**). The platelet function test demonstrated that DAPT effectively inhibited platelet function in both the AA and ADP pathways, and DDAVP treatment was able to restore this platelet dysfunction (**Figure 1E**). Examination of platelet morphology using electron microscopy revealed that DDAVP could reduce the excessive presence of non-functional OCS and increase the levels of reduced α granules in platelets, which were caused by DAPT (**Figure 1F**). Furthermore, using a mouse model of carotid artery blood flow and artery occlusion analysis, it was observed that DDAVP treatment could rescue the delayed or impaired occlusion typically seen with DAPT (**Figure 1G–I**). These findings provide strong support for the ability of DDAVP treatment to alleviate the platelet and coagulation dysfunction induced by antiplatelet therapy based on *in vivo* studies.

**Table 1:** The baseline information of patients in the discovery cohort.

Characteristics	*N*=10
Age (yr)	61 (56–65)
Male	10 (100%)
Reasons for DAPT	
Ischemic stroke	6 (60%)
Coronary artery diseases	2 (20%)
Both of ischemic stroke and coronary artery disease	2 (20%)
Comorbidities	
Hypertension	3 (30%)
Dyslipidemia	4 (40%)
Diabetes mellitus	6 (60%)
Coronary artery diseases	4 (30%)
Ischemic stroke	8 (80%)
Ever-or-now smokers	4 (40%)
Regular drinkers	2 (20%)
Laboratory findings	
Platelet count (×10^9^/L)	221 (179–274)
APTT (s)	27.8 (22.8–33.5)
INR	1.03 (0.99–1.09)
Fibrinogen (g/L)	3.36 (2.73–4.06)

Data are expressed as median (IQR) or number (percentage). APTT: Activated partial thromboplastin time; DAPT: dual antiplatelet therapy; INR: international normalized ratio; IQR: inter-quartile range.

### PRDX5 is associated with the platelet and coagulation dysfunction induced by dual antiplatelet therapy

**Figure 2:**
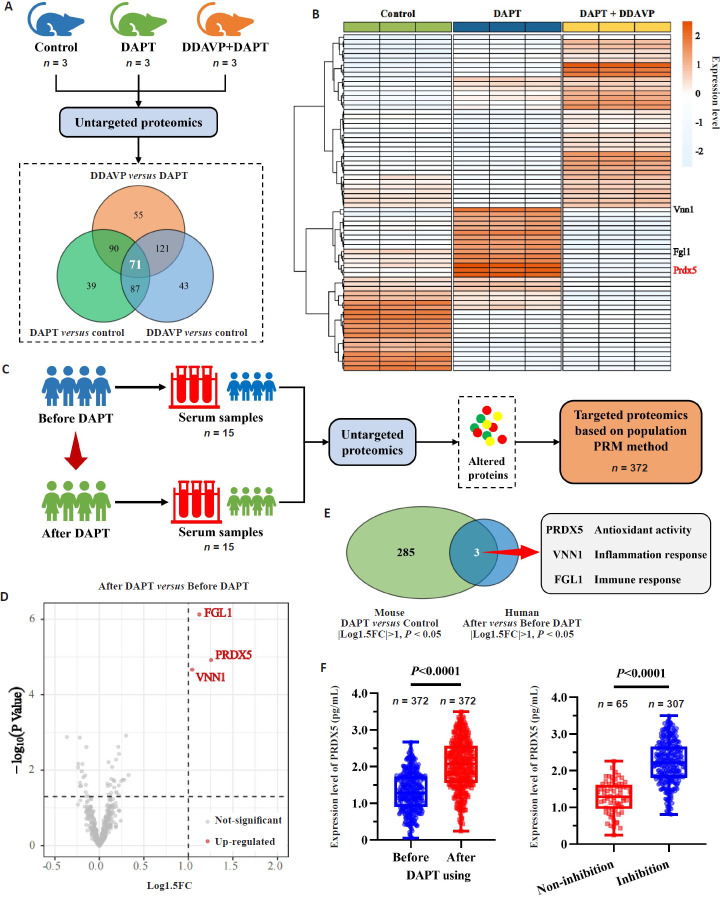
PRDX5 is related to the platelet dysfunction caused by DAPT. (A) The proteomics analysis diagram investigates altered proteins after DAPT and DDAVP treatment, revealing 71 proteins that were altered among the control group, the DAPT group, and the DDAVP + DAPT group. (B) The heatmap plot presents the expression levels of 71 altered proteins among the control group, DAPT group, and DDAVP + DAPT group. PRDX5 was found to be upregulated in the DAPT group compared to both the control group and the DDAVP + DAPT group. Proteins highlighted in red indicate key candidates (including PRDX5) selected for downstream validation, whereas proteins shown in black represent other differentially expressed proteins. (C) The diagram of proteomics analysis to investigate the altered proteins before and after DAPT. Based on a cohort of 15 patients receiving DAPT (derivation cohort), untargeted proteomics analysis revealed altered proteins before and after DAPT. The relationship between altered proteins and DAPT was validated within a population-based cohort of 372 patients receiving DAPT. (D) The volcano plot of untargeted proteomics analysis before and after DAPT treatment, showing that PRDX5 was upregulated after DAPT treatment. (E) The Venn diagram shows the co-dysregulated proteins between mouse models of DAPT and patients before and after DAPT. Three proteins were found to be altered, including PRDX5, which is involved in antioxidant activity. (F) The targeted proteomics analysis within a cohort of 372 patients receiving DAPT (validation cohort). Following DAPT, the expression level of serum PRDX5 was significantly upregulated. Additionally, patients with platelet function inhibition, as indicated by a percentage of AA inhibition > 60% or ADP inhibition > 50%, exhibited a higher serum level of PRDX5 compared to patients without platelet function inhibition. The Wilcoxon signed-rank test was used for paired comparisons (before *vs*. after DAPT), and the Wilcoxon rank-sum test was used for comparisons between two independent groups (inhibition *vs*. non-inhibition). AA: Arachidonic acid; ADP: adenosine diphosphate; DAPT: dual antiplatelet therapy; DDAVP: desmopressin acetate; FC: fold change; PRDX5: peroxiredoxin-5; PRM: parallel reaction monitoring.

To investigate the mechanism by which DDAVP treatment rescues platelet dysfunction induced by DAPT, an untargeted proteomic analysis was conducted on the serum samples obtained from a mouse model of DAPT and DAPT + DDAVP (**Figure 2A**). A total of 327, 316, and 289 altered proteins were identified in the serum samples between the DDAVP + DAPT group and DAPT group, DDAVP + DAPT group and control group, and DAPT group and control group, respectively (**Additional Figure 1A**). The altered proteins in the DDAVP + DAPT group compared with the DAPT group mainly participated in processes related to oxidative stress (**Additional Figure 1B**). Among the identified proteins, 71 were determined to have significant alterations among the control group, DAPT group, and DDAVP + DAPT group (**Figure 2A** and **B**). Notably, PRDX5 was found to function as a protein involved in preventing oxidative stress (**Additional Figure 1C**).

Using a clinical cohort of patients undergoing DAPT, the study investigated the changes in proteins before and after DAPT treatment (**Figure 2C**). The flowchart outlining the enrollment of patients in the validation cohort is presented in **Additional Figure 2A**, while the data generation process is depicted in **Additional Figure 2B**. The characteristics of the patients in the derivation cohort are provided in **Additional Table 1**. Within this cohort, platelet function, measured as the percentage of AA- and ADP-induced inhibition, was significantly increased after DAPT (**Additional Figure 3A**). Untargeted proteomics analysis identified three proteins that exhibited alterations before and after DAPT (**Figure 2D** and **Additional Figure 3B**). Among these three proteins, PRDX5 demonstrated the highest discriminatory ability to distinguish the pre-DAPT condition from the post-DAPT condition (**Additional Figure 3C** and **D**). Further Venn analysis revealed that three proteins, namely PRDX5, VNN1, and FGL1, were identified as co-dysregulated proteins between mouse models undergoing DAPT and patients both before and after DAPT (**Figure 2E**). The clinical data of patients in the validation cohort are presented in **Additional Table 2**. Based on the validation cohort, targeted proteomics analysis demonstrated a significant upregulation of serum PRDX5 following DAPT and in patients exhibiting platelet function inhibition (**Figure 2F**). However, no significant differences were observed in VNN1 and FGL1 levels before and after DAPT, nor between patients with and without platelet function inhibition (**Additional Figure 3E** and **F**).

**Additional Table 1:** The baseline information of patients in the derivation cohort

Characteristics	*N*=15
Age (yr)	53 (49−60)
Male	8 (53%)
Comorbidities	
Hypertension	7 (47%)
Dyslipidemia	1 (7%)
Diabetes mellitus	1 (7%)
Coronary artery diseases	2 (13%)
Ischemic stroke or TIA	7 (47%)
Ever-or-now smokers	5 (33%)
Regular drinkers	3 (20%)
Laboratory findings	
Platelet count (×10^9^)	223 (211−254)
APTT (s)	29.5 (25.3−34.5)
INR	1.01 (0.98−1.04)
Fibrinogen (g/L)	3.08 (2.68−3.85)

Data are expressed as median (IQR) or number (percentage). APTT: Activated partial thromboplastin time; INR: international normalized ratio; IQR: inter-quartile range; TIA: transient ischemic attack.

**Additional Table 2:** The baseline information of patients in the validation cohort

Characteristics	*N*=372
Age (yr)	55 (49−62)
Male	177 (47.6%)
Comorbidities	
Hypertension	142 (38.2%)
Dyslipidemia	35 (9.4%)
Diabetes mellitus	105 (28.2%)
Coronary artery diseases	31 (8.3%)
Ischemic stroke or TIA	195 (52.4%)
Ever-or-now smokers	133 (35.8%)
Regular drinkers	34 (9.1%)
Laboratory findings	
Platelet count (×10^9^)	216 (206−235)
APTT (s)	24.8 (24.4−30.4)
INR	1.00 (0.97−1.05)
Fibrinogen (g/L)	2.80 (2.36−3.50)

Data are expressed as median (IQR) or number (percentage). APTT: Activated partial thromboplastin time; INR: international normalized ratio; IQR: inter-quartile range; TIA: transient ischemic attack.

### Knockout and pharmacological inhibition of PRDX5 rescue the platelet and coagulation dysfunction caused by dual antiplatelet therapy

**Figure 3:**
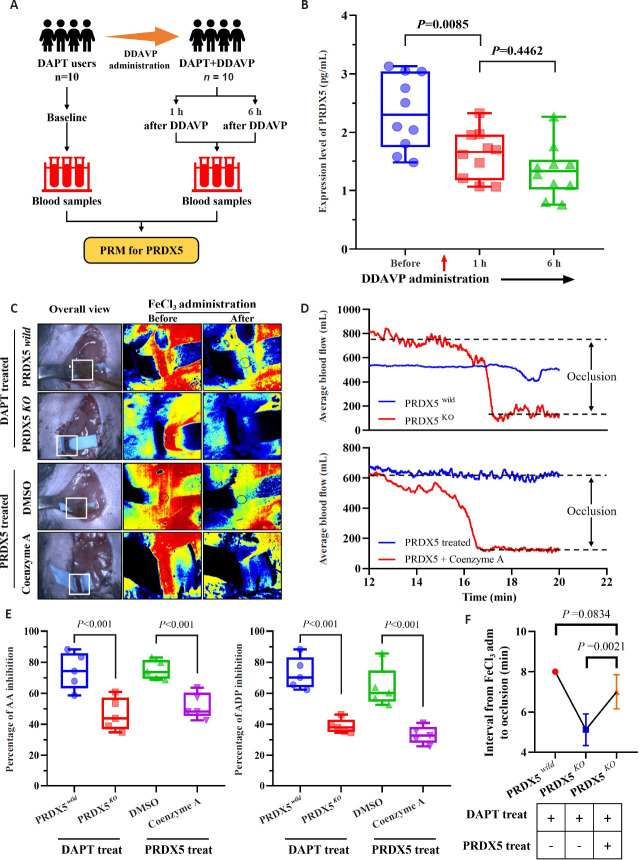
PRDX5 induces platelet and coagulation dysfunction. (A) The diagram illustrates a self-control study conducted to validate the changes in PRDX5 before and after DDAVP treatment (0.4 μg/kg) among patients on DAPT. (B) The targeted proteomics analysis, which show that the expression level of PRDX5 is significantly decreased in the 1^st^ hour after DDAVP treatment. However, there is no significant difference between the expression level of PRDX5 at the 1^st^ and 6^th^ hours after DDAVP treatment. Differences in PRDX5 levels at different time points after DDAVP treatment were analyzed using the Friedman test followed by Dunn’s multiple comparisons test. (C) The images of laser speckle contrast imaging analysis of carotid artery blood flow in each group. The knockout of PRDX5 was found to significantly decrease the anti-coagulation effect of DAPT. Furthermore, the inhibitor of PRDX5 (coenzyme) was shown to suppress the anti-coagulation effect of PRDX5 treatment. (D) The line plot of the carotid artery blood flow after FeCl_3_ administration. It was observed that the knockout of PRDX5 (*n* = 4) resulted in a shortened interval from FeCl_3_ administration to occlusion after DAPT, in comparison to PRDX5^wild^ mice (*n* = 4). Additionally, the inhibitor of PRDX5 (coenzyme, *n* = 4) was shown to prevent the non-occlusion condition caused by PRDX5 supplementation (*n* = 4). (E) The platelet function examination before and after DAPT administration. It is evident that the percentages of AA and ADP inhibition are both significantly increased after PRDX5 supplementation. Conversely, these percentages are significantly decreased after Coenzyme A administration. For each treatment condition, comparisons between two independent groups were performed using the Wilcoxon rank–sum test (Mann–Whitney *U* test). Specifically, PRDX5 wild type and PRDX5 KO were compared under DAPT treatment, and DMSO and Coenzyme A were compared under PRDX5 treatment. (F) The time interval from FeCl_3_ administration to occlusion. It is observed that the knockout of PRDX5 can shorten this interval after DAPT compared to PRDX5^wild^ mice. However, this effect can be rescued by PRDX5 supplementation. Comparisons among independent groups were performed using the Kruskal–Wallis test followed by Dunn’s multiple comparisons test. AA: Arachidonic acid; ADP: adenosine diphosphate; DAPT: dual antiplatelet therapy; DDAVP: desmopressin acetate; DMSO: dimethyl sulfoxide; FeCl_3_: ferric chloride; KO: knockout; OCS: open canalicular system; PRDX5: peroxiredoxin-5; TEM: transmission electron microscopic.

In order to further explore the correlation between DAPT, DDAVP, and PRDX5, this study examined the changes in serum PRDX5 following DDAVP treatment within the discovery cohort of patients receiving DAPT (**Figure 3A**). Following DDAVP treatment, there was a significant decrease in PRDX5 levels in the 1^st^ hour compared to pre-DDAVP treatment conditions; however, no difference was observed in PRDX5 expression between the 1^st^ hour and the 6^th^ hour after DDAVP treatment (**Figure 3B**). These findings suggest that PRDX5 may play a crucial role in platelet dysfunction.

This study delved further into the biological impact of PRDX5 on platelet function using a mouse model of carotid artery blood flow. Knockout of PRDX5 was found to reverse the delayed occlusion caused by DAPT, while supplementation of PRDX5 resulted in delayed artery occlusion. Additionally, pharmacological inhibition of PRDX5 could prevent delayed artery occlusions caused by PRDX5 (**Figure 3C** and **D**, and **Additional Figure 4A**). Platelet function analysis (**Figure 3E**) and platelet morphology analysis (**Additional Figure 4B**) confirmed that knockout of PRDX5 and pharmacological inhibition of PRDX5 were able to restore platelet function, evidenced by a significant decrease in the inhibition of platelet function, an increase in functional OCSs, and an increase in α granules. Moreover, the supplementation of PRDX5 could counteract the effects of PRDX5 deficiency on artery occlusion and platelet function (**Figure 3F** and **Additional Figure 4C**). Hence, based on these findings, it is suggested that DDAVP may mitigate platelet dysfunction by suppressing the expression of PRDX5.

### Desmopressin acetate suppresses the expression of PRDX5 by inducing oxidation in the liver

**Figure 4:**
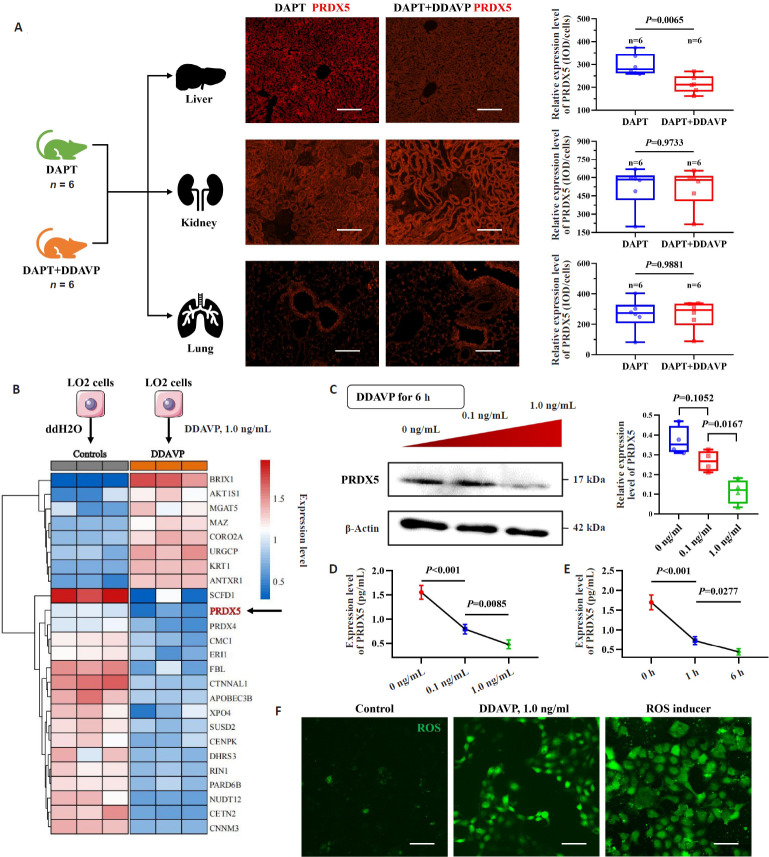
Altered PRDX5 is expressed by liver and could be inhibited by DDAVP. (A) Representative immunofluorescence images and corresponding box plots show PRDX5 expression levels in liver, kidney, and lung tissues from mice treated with DAPT or DAPT + DDAVP (*n* = 6 per group). PRDX5 expression in liver tissue was significantly decreased in the DAPT + DDAVP group, whereas no significant differences were observed in kidney or lung tissues. Comparisons between independent groups were performed using the Wilcoxon rank-sum test (two-sided). Scale bars: 50 μm. (B) The heatmap displays the result of proteomics analysis on LO2 cells before and after DDAVP treatment. It reveals that PRDX5 was downregulated after DDAVP treatment. (C) The Western blot analysis was conducted to investigate the change in PRDX5 levels in LO2 cells after DDAVP treatment. The results indicate that DDAVP treatment significantly decreases the expression of PRDX5. For the Western blot quantification, within-subject comparisons between LO2 cells treated with DDAVP and the corresponding controls were performed using the Kruskal–Wallis test followed by Dunn’s multiple comparisons test (two-sided). (D) The line plots of secreted PRDX5 after DDAVP treatment at different concentrations for 6 hours. The expression level of secreted PRDX5 was examined using the ELISA method. It is observed that at a concentration of DDAVP of 1.0 ng/mL, the expression level of secreted PRDX5 is the lowest. Between-group comparisons of secreted PRDX5 levels at different DDAVP concentrations were performed using the Kruskal–Wallis test followed by Dunn’s multiple comparisons test (two-sided). (E) The line plots of secreted PRDX5 after DDAVP treatment (1.0 ng/mL) at different timepoints. The expression level of secreted PRDX5 was examined using the ELISA method. It is observed that on the 6^th^ hour after DDAVP treatment, the expression level of secreted PRDX5 is the lowest. Comparisons among independent time-point groups were performed using the Kruskal–Wallis test followed by Dunn’s multiple comparisons test (two-sided). (F) The immunofluorescence images present the expression level of ROS in representative cases. Scale bars: 20 μm. DDAVP can induce an increase in ROS expression. DAPT: Dual antiplatelet therapy; DDAVP: desmopressin acetate; ELISA: enzyme-linked immunosorbent assay; PRDX5: peroxiredoxin-5; ROS: reactive oxygen species.

Subsequently, the current study examined the impact of DDAVP treatment on PRDX5 levels in three organs with high expression of PRDX5 (liver, kidney, and lung) using a mouse model of DAPT (**Figure 4A**). The results revealed a significant reduction in the expression of PRDX5 in the liver after DDAVP treatment (*P* = 0.0065). However, no significant change was observed in the expression of PRDX5 in the kidney (*P* = 0.9733) and lung (*P* = 0.9881) following DDAVP treatment.

Furthermore, an untargeted proteomics analysis was performed on LO2 cells treated with and without DDAVP (**Figure 4B**). The analysis revealed that 26 proteins were significantly altered (**Additional Figure 5A**). Functional enrichment analysis showed that these altered proteins mainly participated in processes related to oxidative stress (**Additional Figure 5B** and **C**). A Western blot analysis confirmed that DDAVP treatment could suppress the expression of PRDX5 (**Figure 4C**). Moreover, the expression level of secreted PRDX5 by LO2 cells gradually decreased with increasing concentration of DDAVP and treatment interval (**Figure 4D** and **E**).

**Figure 5:**
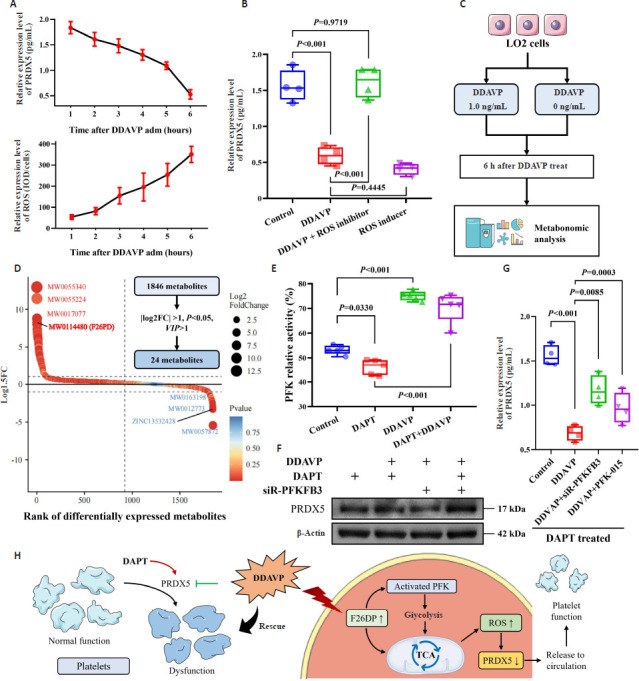
DDAVP inhibits the expression of PRDX5 by promoting glycolysis. (A) The line plots display the time-related changes in PRDX5 and ROS expression after DDVAP treatment. The expression of PRDX5 and ROS shows an opposite trend or exhibits a reverse correlation. Statistical comparisons among groups were performed using the Kruskal–Wallis test followed by Dunn’s multiple comparison test. (B) The the expression level of secreted PRDX5 in each group. The expression level of secreted PRDX5 was measured using the ELISA method. Both DDAVP and the ROS inducer can inhibit the expression of PRDX5. However, when ROS inhibition is applied, it can rescue the decreased PRDX5 caused by DDAVP treatment. Statistical comparisons among groups were performed using the Kruskal–Wallis test followed by Dunn’s multiple comparison test. (C) The diagram illustrates a study investigating the metabolic features of LO2 cells after DDAVP treatment. (D) The ranking dot plot of an untargeted metabonomic study. It identifies 24 significantly altered metabolites out of a pool of 1846 metabolites. (E) The the activity of PFK in each group. DDAVP is shown to enhance the activity of PFK, while DAPT inhibits it. Furthermore, DDAVP is able to counteract the reduced activity of PFK caused by DAPT. Statistical comparisons among groups were performed using the Kruskal–Wallis test followed by Dunn’s multiple comparison test. (F) The Western blot assay was used to investigate the changes in PRDX5. Knockdown or pharmacological inhibition (using PFK-015) of PFKFB3 shows the ability to rescue the decrease in PRDX5 levels caused by DDAVP treatment. (G) The expression level of secreted PRDX5 after different treatments. Knockdown or pharmacological inhibition of PFKFB3 is shown to inhibit the decrease in secreted PRDX5 levels caused by DDAVP treatment. Statistical comparisons among groups were performed using the Kruskal–Wallis test followed by Dunn’s multiple comparison test. (H) The central illustration of PRDX5 in platelet dysfunction induced by DAPT. It shows that DDAVP can rescue this platelet dysfunction by inhibiting the expression of PRDX5. This inhibition is achieved through the promotion of glycolysis and ROS expression. Created with Adobe Illustrator 2024. DAPT: Dual antiplatelet therapy; DDAVP: desmopressin acetate; FC: fold change; PFK: phosphofructokinase 1; PFKFB3: phosphofructokinase-2/fructose-2,6-bisphosphatase 3; PRDX5: peroxiredoxin-5; ROS: reactive oxygen species.

Given that PRDX5 and proteins with significant alterations between mice receiving and not receiving DDAVP treatment are involved in oxidation processes, this study then investigated the expression level of ROS. The results indicated that DDAVP could induce the expression of ROS in LO2 cells (**Figure 4F** and **Additional Figure 5D**). The expression level of ROS exhibited an inverse trend to the expression level of secreted PRDX5 (**Figure 5A**). Furthermore, a ROS inhibitor was able to prevent the decreased expression of secreted PRDX5 caused by DDAVP treatment, while a ROS inducer could inhibit the expression of secreted PRDX5 (**Figure 5B**).

In conclusion, these findings indicate that DDAVP can inhibit the expression of PRDX5 by inducing oxidative stress in the liver.

### Desmopressin acetate inhibits the expression of PRDX5 by promoting glycolysis

Due to the strong connection between metabolism and oxidative stress, an untargeted metabonomic analysis was conducted on LO2 cells treated with and without DDAVP (**Figure 5C**). The analysis revealed that 24 metabolites exhibited significant alterations between the two groups (**Figure 5D** and **Additional Figure 6A**). These altered metabolites were primarily associated with fructose and mannose metabolism (**Additional Figure 6B** and **C**). Among these altered metabolites, fructose-2,6-diphosphate (F26DP) showed the most significant alteration (**Figure 5D** and **Additional Figure 6A**). The difference in F26DP levels between LO2 cells treated with and without DDAVP was confirmed through targeted metabonomic analysis (**Additional Figure 6D**).

Phosphofructokinase 1 (PFK) serves as the pivotal enzyme in the process of glycolysis, and F26DP acts as an allosteric activator of PFK.^30^ This study demonstrated that DAPT could inhibit the activity of PFK, while DDAVP could enhance or restore PFK activity (**Figure 5E**). Importantly, proteomics analysis conducted on LO2 cells treated with and without DDAVP also revealed the upregulation of phosphofructokinase-2/fructose-2,6-bisphosphatase 3 (PFKFB3), an enzyme involved in F26D production, in LO2 cells after DDAVP treatment (**Additional Figure 6E**). Subsequent analysis demonstrated that suppression of PFKFB3 could prevent the decline of PRDX5 in DDAVP-treated LO2 cells (**Figure 5F**). Furthermore, both knockdown and pharmacological inhibition of PFKFB3 were able to increase the expression level of secreted PRDX5 and suppress ROS levels in LO2 cells treated with DDAVP (**Figure 5G** and **Additional Figure 6F** and **G**).

The results of this study revealed the crucial role of PRDX5 in the platelet dysfunction induced by DAPT. Interestingly, DDAVP was found to alleviate this platelet dysfunction by suppressing the expression of PRDX5 through the promotion of glycolysis and oxidation pathways (**Figure 5H**).

## Discussion

DDAVP has been identified as a potential strategy for alleviating platelet and coagulation dysfunction induced by antiplatelet therapy.^6^,^7^,^8^,^31^,^32^,^33^,^34^,^35^ Using a mouse model of DAPT, this study demonstrated that DDAVP effectively rescued the platelet and coagulation dysfunction caused by DAPT. Subsequent proteomics analysis conducted on both the mouse model treated with DDAVP and a clinical cohort of patients receiving either DAPT or DAPT + DDAVP treatment revealed the association of PRDX5 with the rapid restoration of platelet and coagulation function impaired by DAPT. *In vivo* investigations further confirmed that external supplementation of PRDX5 could induce platelet and coagulation dysfunction, thereby hindering the rescue effect of DDAVP on DAPT-induced platelet and coagulation dysfunction. Conversely, knockout of PRDX5 or pharmacological inhibition of PRDX5 exhibited the ability to rescue these dysfunctions. *In vitro* experiments revealed that DDAVP treatment could lead to oxidative stress in liver cells and suppress the expression of PRDX5. Subsequent analyses focusing on metabolic pathways demonstrated that DDAVP could enhance glycolysis and promote the generation of ROS by inducing the expression of PFKFB3. Inhibition of PFKFB3 was found to upregulate the expression of PRDX5 by suppressing glycolysis and reducing ROS levels. These findings suggest that DDAVP has the potential to effectively rescue platelet and coagulation dysfunction caused by antiplatelet therapy.

Compared with earlier studies that mainly evaluated DDAVP as a hemostatic adjunct, our study integrates a DAPT mouse model with a clinical cohort and multi-omics analyses to explore potential mechanisms.^6^,^36^,^37^ In addition to the established von Willebrand factor-related effects of DDAVP, we observed a metabolic–redox pathway involving PFKFB3-driven glycolysis, ROS generation, and PRDX5 regulation that was associated with recovery of platelet and coagulation function under antiplatelet exposure. Moreover, manipulation of PRDX5 levels, including exogenous supplementation, genetic deletion, and pharmacologic inhibition, consistently altered the DDAVP-mediated rescue phenotype, supporting PRDX5 as an important modulator of this response.

While our data strongly support a PFKFB3–glycolysis–ROS–PRDX5 axis downstream of DDAVP, the upstream signaling linking DDAVP to increased PFKFB3 expression remains unclear. As a vasopressin analog, DDAVP primarily activates vasopressin receptors, classically AVPR2 (V2R), to trigger cAMP/PKA signaling, which could plausibly enhance PFKFB3 transcription via CREB-dependent programs. Alternatively, vasopressin receptor signaling can also engage Ca²^+^/PKC and MAPK pathways, which have been implicated in metabolic gene regulation and could contribute to PFKFB3 induction.

Oxidative stress plays a crucial role in platelet activation and the subsequent formation of thrombi.^38^,^39^ Previous studies have demonstrated that oxidative stress and related enzymes promote the process of thrombus formation and amplify platelet activation.^10^,^13^ Mechanistically, oxidative stress can enhance platelet function through various pathways, such as inducing NADPH oxidase and inhibiting glutathione peroxidase, ultimately leading to an increase in ROS and a reduction in platelet aggregation.^11^ DDAVP treatment has been found to induce oxidative stress in liver cells and suppress the expression of antioxidants, which is critical for the rescue effect of DDAVP on platelet dysfunction caused by DAPT.

PRDX5, functioning as an antioxidant, can mitigate oxidative stress and affect platelet function.^19^,^20^ The effects of PRDX5 on platelet function and the influence of DDAVP on PRDX5 can be attributed to the following factors: (1) Platelets have the capacity to take up PRDX5 from the serum, leading to a reduction in intraplatelet ROS levels, thereby potentially decreasing the number of pre-activated platelets. (2) DDAVP treatment can induce oxidative stress by promoting glycolysis. The increased ROS production can deplete PRDX5 levels and limit its release into the circulation, thereby preventing further platelet pre-activation. Acting as an antioxidant, elevated levels of PRDX5 inhibit platelets from transitioning into a pre-activated state. Consequently, when the concentration of PRDX5 is increased, platelet function is restrained.^40^,^41^

The research indicates that DDAVP has the potential to be a viable strategy for rescuing platelet dysfunction in patients receiving antiplatelet therapy who experience emergency situations. Its application is not limited to a particular disease and can be beneficial across various emergency scenarios. Moreover, our findings have positive implications for the healthcare system by potentially reducing the requirement for blood products and transfusions, minimizing the risk of postoperative bleeding and subsequent surgeries, and lowering medical costs. When extrapolating these findings to broader clinical use, DDAVP should be considered with appropriate caution. Clinically, hyponatremia due to water retention is the most relevant safety concern (particularly in emergency settings without strict fluid control), and a potential thrombotic risk may exist in predisposed patients; therefore, fluid restriction/serum sodium monitoring and careful patient selection should accompany any DDAVP-based strategy. However, further clinical investigations and trials are necessary to validate our conclusion.

Despite these findings, several limitations should be acknowledged. First, the clinical cohort size was relatively small, and the translational relevance of the mechanistic observations requires validation in larger, independent patient populations. Second, although our data support involvement of a PFKFB3–glycolysis–ROS–PRDX5 pathway downstream of DDAVP, upstream receptor-mediated signaling and cell-type–specific contributions were not directly interrogated. Finally, while hepatic metabolic responses were highlighted in this study, potential effects in other organs or circulating cells warrant further investigation. Future studies addressing these aspects will be important to fully define the therapeutic scope of DDAVP in antiplatelet-associated platelet dysfunction.

In summary, this preclinical investigation showcased the effectiveness of DDAVP in mitigating platelet dysfunction and coagulation impairments induced by antiplatelet therapy through the inhibition of PRDX5 expression. The rapid induction of oxidative stress in the liver via glycolysis promotion by DDAVP plays a critical role in reducing PRDX5 expression. This preclinical study has the potential to provide innovative theoretical frameworks and therapeutic approaches for the prompt rescue of platelet dysfunction resulting from antiplatelet therapy.

## Additional files:

***Additional file 1:*** *Supplementary methods and extended experimental procedures.*

Additional file 1 Supplementary methods and extended experimental procedures

***Additional Table 1:*** *The baseline information of patients in the derivation cohort.*

***Additional Table 2:*** *The baseline information of patients in the validation cohort.*

***Additional Figure 1:*** *The flowchart of patient enrollment and data generation.*

Additional Figure 1 The flowchart of patient enrollment and data generation.
(A) The flowchart of patient enrollment in the validation cohort. The validation cohort included 372 patients receiving DAPT for UIA embolization from 419 patients. (B) The summary of data generation. (C) The relationship of biological process and altered proteins. PRDX5 could participate in several processes related to oxidative stress. DAPT: Dual antiplatelet therapy; DDAVP: desmopressin acetate; PRDX5: peroxiredoxin-5; TEG: thromboelastography; UIA: unruptured intracranial aneurysms; VNN1: pantetheinase.

***Additional Figure 2:*** *PRDX5 is related to platelet dysfunction caused by DAPT.*

Additional Figure 2 PRDX5 is related to platelet dysfunction caused by DAPT.
(A) The volcano plots of the altered proteins in serums between the mice of DDAVP + DAPT and DAPT groups (327 altered proteins), between the mice of DDAVP + DAPT and control groups (316 altered proteins), and between the mice of DAPT and control groups (289 altered proteins). (B) The enrichment analysis showing the biological process of 337 altered proteins between the mice of DDAVP + DAPT and DAPT groups. Altered proteins mainly participate in the processes related to oxidative stress. DAPT: Dual antiplatelet therapy; DDAVP: desmopressin acetate; FC: fold change.

***Additional Figure 3:*** *PRDX5 is associated with platelet dysfunction caused by DAPT.*

Additional Figure 3 PRDX5 is associated with platelet dysfunction caused by DAPT.
(A) The platelet function examination before and after DAPT. The percentage of AA and ADP inhibition is significantly increased after DAPT. Statistical comparisons between paired groups were performed using the Wilcoxon signed-rank test. (B) The flowchart of identifying the key proteins related to DAPT. Three altered proteins are identified before and after DAPT. (C) The ROC curve showing the performance of altered proteins to identify condition before DAPT and condition after DAPT. (D) The histogram of the performance of altered proteins to identify condition before DAPT and condition after DAPT. ROC curve analysis was performed, and AUC was calculated. Pairwise comparisons of AUCs were conducted using DeLong's test for correlated ROC curves. Exact P values are indicated in the figure. (E) The targeted proteomics analysis based on a cohort of 372 patients receiving DAPT. After DAPT, the expression level of serum VNN1 was significantly upregulated. There is no significant difference in expression of VNN1 between patients without platelet function inhibition (percentage of AA inhibition > 60% or ADP inhibition > 50%) and patients with platelet function inhibition. The Wilcoxon signed-rank test was used for paired comparisons (before vs. after DAPT), and the Wilcoxon rank-sum test was used for comparisons between two independent groups (inhibition vs. non-inhibition). (F) The targeted proteomics analysis based on a cohort of 372 patients receiving DAPT. After DAPT, the expression level of serum FGL1 had no significant difference. There is no significant difference in expression of FGL1 between patients without platelet function inhibition (percentage of AA inhibition = 60% or ADP inhibition = 50%) and patients with platelet function inhibition. The Wilcoxon signed-rank test was used for paired comparisons (before vs. after DAPT), and the Wilcoxon ranksum test was used for comparisons between two independent groups (inhibition vs. non-inhibition). AA: Arachidonic acid channel; ADP: adenosine diphosphate channel; AUC: the area under the curve; DAPT: dual antiplatelet therapy; DDAVP: desmopressin acetate; FC: fold change; FGL1: fibrinogen-like protein 1; PRDX5: peroxiredoxin-5; ROC: receiver operating characteristic; VNN1: pantetheinase.

***Additional Figure 4:*** *DDAVP rescues the platelet dysfunction caused by DAPT and inhibits the expression of PRDX5.*

Additional Figure 4 DDAVP rescues the platelet dysfunction caused by DAPT and inhibits the expression of PRDX5.
(A) The time interval from FeCl_3_ administration to occlusion. The knockoff of PRDX5 can shorten the interval from FeCl_3_ administration to occlusion after DAPT, compared with PRDX5^wild^ mice. The inhibitor of PRDX5 (coenzyme) can prevent the no occlusion condition caused by PRDX5. For each treatment condition, comparisons between two independent groups were performed using the Wilcoxon rank-sum test (Mann–Whitney *U* test). Specifically, PRDX5 wild type and PRDX5 KO were compared under DAPT treatment, and DMSO and Coenzyme A were compared under PRDX5 treatment. (B) The representative TEM images of platelets in each group. After being treated by DDAVP, platelets showed a restorative OCS and increasing α granules, compared with the control group (DAPT treatment). After supplementing PRDX5, the platelets have significantly fewer closed OCSs and α granules. After coenzyme A administration, the platelets show functional OCSs and increasing α granules. Yellow arrows indicate OCS, green arrows indicate closed OCS (nonfunctional OCS), and white arrows indicate α granules. Scale bars: 500 nm. (C) The platelet function examination before and after DAPT administration. The percentages of AA and ADP inhibition are both significantly increased after PRDX5 knockoff comparing with PRDX5^wild^ mice, which could be rescued by PRDX5 supplementation. Statistical comparisons among groups were performed using the Kruskal–Wallis test followed by Dunn's multiple comparison test. AA: Arachidonic acid channel; ADP: adenosine diphosphate channel; DAPT: dual antiplatelet therapy; DDAVP: desmopressin acetate; DMSO: dimethyl sulfoxide; FeCl_3_: ferric chloride; KO: knockout; OCS: open canalicular system; PRDX5: peroxiredoxin-5; TEM: transmission electron microscopic.

***Additional Figure 5:*** *Altered PRDX5 is expressed by the liver.*

Additional Figure 5 Altered PRDX5 is expressed by the liver.
(A) The volcano plots of altered proteins between LO2 cells treated with DDAVP and controls. PRDX5 is significantly ecreased in liver cells (LO2 cells) treated by DDAVP. (B) The bubble plot of enrichment analysis. Altered proteins mainly participate in the processes related to oxidative stress. (C) The Sankey plot of the relationship between altered proteins and biological processes. PRDX5 can participate in several processes related to oxidative stress. (D) Relative ROS expression levels. Statistical comparisons among groups were performed using the Kruskal–Wallis test followed by Dunn's multiple comparison test. Exact *P* values are indicated in the figure. DDAVP can induce the expression of ROS. DAPT: Dual antiplatelet therapy; DDAVP: desmopressin acetate; PRDX5: peroxiredoxin-5; ROS: reactive oxygen species.

***Additional Figure 6:*** *DDAVP inhibits the expression of PRDX5 by promoting glycolysis.*

Additional Figure 6 DDAVP inhibits the expression of PRDX5 by promoting glycolysis.
(A) The dot plot of the VIP value of 24 altered metabolites between LO2 cells treated with DDAVP and controls. (B) The pie plot of the class of altered metabolites between LO2 cells treated and not treated by DDAVP. (C) The bubble plot of enrichment analysis of altered metabolites treated and not treated by DDAVP. The fructose metabolism is related to DDAVP treatment. (D) The histogram of targeted metabolomics analysis of F26DP. (E) PFKFB3 is the key to the generation of F26DP. Statistical comparisons between two independent groups were performed using the Wilcoxon rank–sum test. (F) The representative of immunofluorescence studies to investigate the change of ROS after DDAVP administration. Knockdown or pharmacological inhibition (PFK-015, a specific inhibitor for PFKFB3) of PFKFB3 can inhibit the increasing expression of ROS caused by DDAVP. Scale bars: 10 μm. (G) Relative ROS expression levels (upper panel) and relative PRDX5 mRNA levels (lower panel). Statistical comparisons among groups were performed using the Kruskal–Wallis test followed by Dunn's multiple comparison test. Exact *P* values are indicated in the figure. DDAVP: Desmopressin acetate; DAPT: dual antiplatelet therapy; DMSO: dimethyl sulfoxide; F26DP: fructose-2:6-diphosphate; PFKFB3: phosphofructokinase-2/fructose-2,6-bisphosphatase 3; PFK1: phosphofructokinase 1; PRDX5: peroxiredoxin-5; ROS: reactive oxygen species; VIP: variable importance in projection.

## Data Availability

*All relevant data are within the paper and its Additional files.*
