# Supplementary material for: Glycolysis-dependent reactive oxygen species mediate desmopressin acetate–induced rescue of platelet dysfunction caused by antiplatelet therapy
Source: Med Gas Res. 2026 Mar 26;16(4):323–33. doi: 10.4103/mgr.MEDGASRES-D-25-00353 (PMC13456463; doi:10.4103/mgr.MEDGASRES-D-25-00353)
Supplement: Supplementary file 1 [file MGR-16-323_Suppl1.pdf]

Additional file 1 Supplementary methods and extended experimental procedures

Study population

The discovery cohort recruited patients who received standard dual antiplatelet therapy (DAPT; aspirin 100 mg once daily plus clopidogrel 75 mg once daily) from November 2021 to December 2021. The inclusion and exclusion criteria included: (1) male, aged 18−75 years; (2) had a history of long-term oral DAPT, defined as continuous administration for over 1 week before the DDAVP injection. Exclusion criteria included: (1) patients with a history of thrombocytopenia, coagulopathy, or other hematologic disorders; (2) patients with known contraindications to DDAVP therapy.

The derivation cohort prospectively enrolled 15 patients who received DAPT because of planned stent-assisted coiling for unruptured intracranial aneurysms at Beijing Tiantan Hospital, from January 2022 to August 2022. The inclusion and exclusion criteria were listed as follows: (1) received stent-assisted coiling for unruptured intracranial aneurysms; (2) aged 18−75 years; (3) with a modified Rankin scale score ≤ 2.[1] We then excluded patients who: (1) had compilations related to the surgical accidents and quality of the material, such as coil hesitation and incompletely opened stents; (2) received simultaneous treatment for other cerebrovascular diseases, such as arteriovenous malformations; (3) could not bear dual antiplatelet therapy (aspirin plus clopidogrel), because of an allergy to aspirin or clopidogrel; (4) aneurysms were recurrent or incompletely embolized; (5) received postoperative prophylactic use of glycoprotein IIb/IIIa antagonists (e.g., tirofiban); (6) had malignant diseases, such as malignant tumors, renal failure or hepatic failure; (7) took medications that interfere with aspirin absorption and metabolism; (8) were with poor medication compliance (Morisky Medication Adherence Scale < 6).

The validation cohort also prospectively included patients receiving DAPT because of planned stent-assisted coiling for unruptured intracranial aneurysms from July 2022 to June 2023. The inclusion and exclusion criteria were the same as those of the discovery cohort.

Mouse model of DAPT and DDAVP treatment

The model was established using C57BL/6N mice. Female C57BL/6J mice, aged 8 weeks and weighing between 18 to 22 g, were purchased from Gempharmatech Co., Ltd. (Nanjing, China). The protocol of animal studies was approved by the Animal Care and Use Committee of Chinese Neurosurgical Institution (approval No. 202103005; March 6, 2023). This study was conducted in compliance with the National Institutes of Health Guide for the Care and Use of Laboratory Animal*s*. Mice were accommodated in a room maintained at a temperature of 25 ± 2°C and a relative humidity of 50 ± 5%. Mice were subjected to a 12-hour dark/light cycle and had unrestricted access to both food and water. Before any experimental procedures, mice underwent an acclimatization period of at least 1 week. The DAPT mouse model was established by administering aspirin (HY-14654, MCE, Shanghai, China) and clopidogrel (HY-15283, MCE). For administration, aspirin and clopidogrel were prepared in corn oil, respectively. Mice were administered 20 μg/kg aspirin (once daily) and 15 μg/kg clopidogrel (once daily), via oral gavage for 7 days.

Mouse model of DDAVP therapy was established based on the mouse model of DAPT. After an appropriate DAPT, mice would receive an intraperitoneal injection of 3.6 μg/kg DDAVP (P9050-5mg; Beyotime Biotechnology, Shanghai, China). One hour later, these mice were used for further analysis.

Cell culture and treatment

LO2 cells were purchased from the American Type Culture Collection (Manassas, VA, USA). Cells were cultured with Dulbecco's modified Eagle medium containing 10% fetal bovine serum (Gibco, Thermo, Sydney, NSW, Australia), and maintained at 37°C, 5% CO2, and saturated humidity.

Before treatment, cells were starved for 24 hours. Then, cells were treated with 1.0 ng/mL of DDAVP and deionized water for 6 hours, respectively. The cells in stable growth were used in the following experiments.

Proteomics and functional analysis

Frozen mouse liver tissue and LO2 cells were homogenized in radioimmunoprecipitation assay buffer (Sigma-Aldrich, St. Louis, MO, USA), protease and phosphatase inhibitors (Sobarbio, Beijing, China). The lysis buffers were placed on ice for 30 minutes and then centrifuged for 20 minutes at 15,000 × *g*. The supernatant was collected, and the concentration of the extracted protein was quantitatively analysed using the bicinchoninic acid method (missing citation). Samples with total proteins < 150 μg were pooled to ensure sufficient proteins for further analysis by liquid chromatography with tandem mass spectrometry (LC-MS/MS). A total of 150 μg of each sample was used for proteomic analysis. LC-MS/MS analysis was performed using the isobaric tags for relative and absolute quantitation (iTRAQ) method (Wayenbio Corporation, Shanghai, China).

The raw data were screened using Proteome Discoverer (Version 1.4, Thermo Fisher Scientific, Waltham, MA, USA, https://www.thermofisher.cn/cn/zh/home/industrial/mass-spectrometry/liquid-chromatography-mass-spectrometry-lc-ms/lc-ms-software/multi-omics-data-analysis/proteome-discoverer-software.html?utm_source=chatgpt.com).

Any proteins with a unique peptide of 0 or that were described as false positive proteins in the Decoy database were excluded. Differential expression analysis was performed using R software (version 3.6.2; R Foundation for Statistical Computing, Vienna, Austria; https://www.r-project.org). The fold change ratio (FC) was calculated as logFC = log2, and the *P* value was calculated as −log10. In proteomics analysis, significant difference between the two groups of proteins is defined as those | log2 (FC) | > 1, the VIP > 1 and *P* values < 0.05. Venn analyses were performed to identify the intersection of the groups using R with Venn (version 0.1.0; https://cran.r-project.org/package=Venn). The proteins at the intersection of the three groups were used for further bioinformatics analysis via CytoScape (version 3.6.1; Cytoscape Consortium; https://cytoscape.org). Using ClueGo (a plug-in in CytoScape), pathway enrichment analysis was performed based on the Kyoto Encyclopaedia of Genes and Genomes (KEGG) database (KEGG; https://www.kegg.jp), and biological process (BP) and cellular components (CC) were performed based on the Gene Ontology (GO) database (http://geneontology.org). The results of enrichment analyses were plotted using R software with the packages (Heatmap (version 0.2.3; https://cran.r-project.org/package=pheatmap), Volcano (https://bioconductor.org/packages/EnhancedVolcano), Barplot colour group (v 0.1.0, https://hiplot.cn/), Bubble (v 0.1.2, https://hiplot.cn/), and Gene ranking dotplot (v 0.1.0, https://hiplot.cn/).

Parallel reaction monitoring analysis

The peptides were subjected to NSI source followed by tandem mass spectrometry (MS/MS) in Q Exactive™ Plus (Thermo) coupled online to the UPLC. Peptides were then selected for MS/MS using NCE setting as 27 and the fragments were detected in the Orbitrap (Thermo Fisher Scientific, Waltham, MA, USA, Cat# IQLAAEGAAPFALGMBDK) at a resolution of 17,500. A data-independent procedure that alternated between one MS scan followed by 20 MS/MS scans. Automatic gain control was set at 3E6 for full MS and 1E5 for MS/MS. The maximum IT was set at 20 ms for full MS and auto for MS/MS. The isolation window for MS/MS was set at 2.0 m/z. The resulting MS data were processed using Skyline (version 3.6; MacCoss Lab Software, University of Washington, Seattle, WA, USA; https://skyline.ms). Peptide settings: enzyme was set as Trypsin [KR/P], Max missed cleavage set as 2. The peptide length was set as 8−25, Variable modification was set as Carbamidomethyl on Cys and oxidation on Met, and max variable modifications was set as 3. Transition settings: precursor charges were set as 2, 3, ion charges were set as 1, 2, ion types were set as b, y, p. The product ions were set as from ion 3 to last ion, the ion match tolerance was set as 0.02 Da.

Mouse model of carotid artery blood flow and laser speckle contrast imaging

A mouse model of carotid artery blood flow was established by using ferric chloride ($FeCl_3$)-induced injury model.

Mice were anesthetized using avertin (tribromoethanol, 1.25% w/v, 25 mg/kg, intraperitoneal injection, Cat# IR9183, Solabio, Beijing, China) via intraperitoneal injection. Following this, they were securely positioned in a supine orientation on a heating pad maintained at 37°C to ensure body temperature stability. A precise incision was made to expose the skin overlying the common carotid artery. Careful blunt dissection was then employed to remove the fascia, subsequently exposing a segment of the artery. Once prepared, the mice were carefully positioned under the RFLS III (Full-Field Laser Perfusion Imager; RWD Life Sciences, Shenzhen, China). Proper alignment ensured the target area was aptly placed beneath the laser. For thrombosis induction, a 20% ferric chloride solution was applied to the exposed artery. After 5 minutes, the filter paper was used to delicately absorb the ferric chloride solution. Blood flow through the artery was then continuously monitored and documented for a 20-minute window using the Laser Speckle Contrast Imaging camera. Throughout this observation phase, the distance between the Laser Speckle Contrast Imaging camera and the subject was meticulously maintained between 20 and 25 cm.

The RFLS III system was employed for both thrombosis visualization and blood flow assessment. Once the carotid artery was exposed, the mice were positioned within the RFLS III's observation zone. We utilized the multi-image measurement mode to ensure continuous observation of blood flow, making certain that the entirety of the exposed carotid artery remained centered within the observation zone as displayed on the computer monitor. It was essential to adjust the color palette for optimal visualization of the target area.

Subsequent observations of blood flow were made at 2-second intervals for a total of 20 minutes. The blood flow imagery of the carotid artery was represented using arbitrary units, visualized through a 256-color palette via the RFLS III software. Real-time flux data were procured and served as the primary dataset, processed using the RFLS III measurement software (Version 5.0).

Blood flow within the designated regions of interest of the carotid artery was meticulously analyzed. This analysis produced a graphical representation illustrating the variations in blood flow over time. Using this graph, the time to occlusion was discerned. Specifically, this duration was defined as the interval from the moment the ferric chloride solution was removed to the cessation of blood flow in the artery. Should occlusion not manifest within 20 minutes post-injury, a standard time of 20 minutes should be employed for subsequent statistical analysis.

Light transmission aggregometry

For mice, blood is collected via the retro-orbital sinus after anesthesia with avertin (25 mg/kg, intraperitoneal injection). The collected blood is stored in an anticoagulant tube.

For platelet-rich plasma (PRP) preparation, centrifuge the collected blood at room temperature for 10 minutes at $100 \times g$. Following this, separate and transfer the top two-thirds of the plasma, which is platelet-enriched, into clean tubes. To produce platelet-poor plasma (PPP), centrifuge the same tube again for 2 minutes at $11{,}000 \times g$. The resultant supernatant is the PPP, which should be transferred to a new tube. Using an appropriate amount of PPP, resuspend the pellet to obtain the PRP. The platelet concentration in PRP can be adjusted to 1×10^8 platelets/μL using the PPP, as measured by a hemocytometer (Improved Neubauer chamber, 0.1-mm depth; CitoTest, Jiangsu Shitai Laboratory Equipment Co., Ltd., Nanjing, China).

Upon switching on the aggregometer, the PPP baseline is set using empty channels. A tube with only a small magnet is used to establish the PRP baseline. Before introducing the agonist solution, the baseline tracings for light transmission aggregometry on PRP should be stable for at least a minute. The aggregation analysis is carried out at 37°C. Using ADP and arachinonic acid as agonists. A small disposable magnet is placed in each tube. The PRP is incubated at 37°C for 2 minutes. For ADP, 290 μL PRP is added to each test tube and 10 μL of each agonist solution is added to the tubes with PRP by the use of a long pipette (one agonist per tube). For aarachinonic acid, 270 μL PRP is added to a test tube and 30 μL of the agonist solution is added to the tubes with PRP by the use of a long pipette (one agonist per tube). The aggregation curve is then monitored for 5 minutes. Initially, a control sample is used to ensure accurate testing. All analyses should be completed within 4 hours of blood sampling to avoid inaccurate

results due to reduced platelet activity.

Transmission electron microscopy

Pre-fixation of platelet concentrate (PC) was carried out by mixing 2 mL PC with 8 mL of a 10% buffered formaldehyde solution (BD CellFIX™, Becton Dickinson, Vienna, Austria) in order to avoid shape change of platelets by the subsequent preparation steps. Immediately after pre-fixation, the formaldehyde-pre-fixed PC were centrifuged at 800 × *g* for 10 minutes at 22°C. After discarding the supernatant, the pellet was fixed with 10 mL of 2.5% glutaraldehyde (Fluka, Vienna, Austria) in cacodylate buffer, pH 7.2, for 90 minutes at 4°C. After fixation, platelets were washed twice by centrifugation at 800 × *g* for 10 minutes at 4°C and transferred to 2 mL Eppendorf vials (Eppendorf AG, Hamburg, Germany), pelleted again and fixed with 1% OsO_4 (Fluka) for 90 minutes. The pellets were dehydrated in a graded series of ethanol (50%, 70%, 90%, 96% and 100%) and embedded in Epon (Serva, Heidelberg, Germany). Sections (70–80 nm) of Epon-embedded platelets were cut with an UltraCut-UCT ultramicrotome (Leica Inc., Vienna, Austria), transferred to copper grids, and routinely stained with uranyl acetate and lead citrate.

The sections were viewed under an EM 900 transmission electron microscope (Carl Zeiss, Oberkochen, Germany), equipped with a 1k wide-angle slow-scan CCD camera, allowing distortion-free images for photomontages (Tröndle, Munich, Germany) at 80 kV. Panorama views consisting of eight single digital images at a magnification of 3000×, providing a resolution of about 3700 × 1800 pixels. The software allows an exact alignment and stitching of the single image.

Immunofluorescence

The mouse tissue paraffin sections with appropriate volumes were taken and subjected to dehydration, fixation, and paraffin embedding. For immunofluorescence, sections were blocked with Protein Block (Abcam, Cambridge, UK) after antigen retrieval (by sodium citrate or ethylene diamine tetraacetic acid) and permeabilization. The sections were then incubated with primary antibodies at 4°C overnight. Next, the sections were incubated with a secondary antibody. The sections were then stained with 4',6-diamidino-2-phenylindole (Solabio).

ROS was detected by a chemical fluorescence probe method using 2',7'-dichlorofluorescein diacetate (DCFH-DA; Sigma-Aldrich) as a fluorescent probe for ROS detection. First, the probe was prepared with dimethyl sulfoxide as a stock solution at a concentration of 10 mM and subsequently diluted to 20 μM as the working solution used in the experiments. This working solution was added to the cell culture dish and incubated with the cells for 30 minutes to prompt the probe to penetrate the cell membrane and enter the cell interior. Upon completion of incubation, cells were washed with sterile phosphate-buffered saline (PBS) to remove DCFH-DA that failed to enter the cells. Subsequently, the green fluorescence produced in the cells was observed and recorded by fluorescence microscopy (BX53, Olympus Corporation, Tokyo, Japan) and detected using the fluorescein isothiocyanate (FITC) filter group. The changes in ROS levels can be quantitatively assessed by comparing the difference in fluorescence intensity between the treated group and the control group (the untreated cells).

A laser confocal microscopy workstation (LSM 710, ZEISS, Jena, Germany) was used to capture images. Identical conditions and set integration times were applied to facilitate comparisons between samples.

The ImageJ software (version 1.8.0) was used to calculate the integrated optical density and count the cell number based on the 4',6-diamidino-2-phenylindole image. We used the integrated optical density/cells for further analysis.

Western blot analysis

LO2 cells were homogenized in radioimmunoprecipitation assay buffer and mixed with protease inhibitors and phosphatase inhibitors. After digestion and centrifugation, all the visible components were removed. The bicinchoninic acid method was employed to determine the protein concentration of the lysates. Equal amounts of

protein were separated by sodium dodecyl sulfate polyacrylamide gel electrophoresis and electro-transferred to polyvinylidene difluoride membranes. Subsequently, membranes were blocked with PBS plus Tween 20 containing 5% bovine serum albumin (Sigma-Aldrich) for 1 hour and then probed with primary antibodies at 4°C overnight. After primary incubation and washing with PBS plus Tween 20, the membranes were incubated with secondary antibodies for 1 hour at room temperature. Bands were visualized using an enhanced chemiluminescence detection system (GeneSys, Gene Corporation, Syngene, Cambridge, UK). The expression levels were quantified using ImageJ software. The expression levels of target proteins were evaluated using densitometric analysis. Ratios of target protein densitometric measurements of β-actin were used as controls. Anti-PRDX5 rabbit monoclonal antibody (Abcam, Cambridge, UK; Cat# ab180587, RRID: AB_2904214; dilution: WB 1:1000, IF 1:200). Anti-β-actin rabbit monoclonal antibody (ABclonal, Wuhan, China; Cat# AC038; RRID: AB_2863784; dilution: WB 1:5000). Goat anti-Rabbit IgG (H+L), HRP-conjugated (Cell Signaling Technology, Shanghai, China; Cat# 7074; RRID: AB_2099233; dilution: WB 1:1000). Goat anti-Rabbit IgG (H+L) Cross-Adsorbed, Alexa Fluor 594–conjugated (Thermo Fisher Scientific/Invitrogen; Cat# A-11012; RRID: AB_2534079; dilution: IF 1:500).

Metabonomic analysis

The method for harvesting adherent cells is as follows: Begin by removing the culture medium and discarding the growth medium. Subsequently, rapidly rinse the cells 2 to 3 times with PBS pre-chilled to 4°C. Thereafter, add a small amount of PBS and gently scrape off the cells using a cell scraper, transferring them into a centrifuge tube. Centrifuge at a low speed of 1000 × *g* for 30 seconds at 4°C, and then proceed with cell count. Sample with a concentration of 1×10^7 cells per sample, and transfer the cell suspension into a 2 mL collection centrifuge tube. Wash the cells again 2 to 3 times with PBS pre-chilled to 4°C. Then, centrifuge at a low speed of 1000 × *g* for 10 minutes at 4°C and discard the supernatant. Subsequently, rapidly freeze the samples in liquid nitrogen for 15 minutes, and then store them at −80°C.

Untargeted metabolite screening was performed on a Q-TOF tandem mass spectrometer (Triple TOF 5600TM, AB SCIEX Co., Framingham, MA, USA), equipped with ESI and APCI ion sources in the positive/negative-ion mode through an Analyst TF 1.7.1 data processing system. Chromatography was performed on a Shimadzu prominence system with a binary pump, an online degasser, an autosampler and a column oven (Shimadzu Scientific Instruments, INC., Columbia, WA, USA). The separation was achieved in a Waters ACQUITY HSS T3 column (2.1 × 100 mm, 1.8 μm) at 40°C. Gradient elution was programmed in a mobile phase consisting of formic acid in 0.1% (v/v) water (A) and acetonitrile (B) at a flow rate of 250 μL/min as follows: 0−5 minutes, 2−60% B (v/v); 5−10 minutes, 60% (v/v); 10−17 minutes, 60−100% B (v/v); 17−20 minutes, 100% B. The system was returned to the initial conditions in 0.1 minutes and was re-equilibrated for 5 minutes. The autosampler temperature was set at 4°C and the injection volume was 2 μL in the positive-ion mode as well as 5 μL in the negative-ion mode. The LC-MS data wasacquired in both the positive- and negative-ion mode. Detailed parameters were as follows: spray voltage 5.5 kV or −4.5 kV, declustering voltage 80 V or −80 V, vaporizer temperature 450°C, turbo gas 50psi, nebulizer gas 55 psi and curtain gas 35 psi. A fullscan analysis was performed in the TOF mode with a scan range of 50-1000 m/z, and aMS/MS analysis was accomplished in the information-dependent acquisition (IDA) mode with a collision energy (CE) of 45/30/15 or −45/−30/−15 eV. Acquired data wasauto-calibrated according to the calibration manufacturer's guidelines. Analyst TF 1.7.1 (Sciex) was used for data acquisition and processing. External mass calibration was performed on every five sample injections. Plasma and tissue samples were randomized in the sequence, QC and mixed-standard samples were also analyzed repeatedly within the analytical run on every 10 samples to evaluate their chromatographic reproducibility. The obtained raw spectrogram was processed using Progenesis QI (Waters Milford Co., Milford, MA, USA). Metabolites were identified at 2 levels: 1) accurate mass matching, and 2) MS/MS confirmation. Mass tolerance for database search was 5 ppm for MS and 15 ppm for MS/MS. The instrument stability was monitored using QC samples. The intensity of extracted variables was normalized to the total area to reduce the

variations caused by sample injections and enrichment factors. After peak deconvolution, alignment, integration and normalization, a table containing the retention time, exact mass pairs and normalized intensity of each variable wasobtained for a multivariate statistical analysis.

Potential biomarker candidates were selected from project (VIP) > 1, S-plot, and the raw data plot in orthogonal partial least-square discriminant analysis (OPLS-DA) model, as well as an independent *t*-test ($P < 0.05$) based on variable importance. Finally, the fragments, isotopes and adduct ions were manually removed according to corresponding extracted ion chromatograms (XICs). Briefly, the [M+H] + or [M-H]- ions of the metabolites were obtained through high-resolution mass spectrometry, meanwhile a possible molecular composition of the metabolites was obtained based on the accurate mass of molecular ions and the isotope abundance ratio. To obtain structural information, we conducted a LC-MS/MS spectrum analysis and performed a public mass spectrum library search using the resulting MS/MS spectra as queries. Possible structures were inferred through database search, including HMDB (http://hmdb.ca), Massbank (https://massbank.eu/MassBank/) and METLIN (https://metlin.scripps.edu) with exact molecular weights. In addition, the retention time and fragmentation characteristic ions were obtained through a LC-MS/MS analysis of commercial standards, and a comparative analysis through progenesis QI software (WatersMilford, MA, USA) was used to finally confirm the structure of the metabolites.

Reference

1. Saver JL, Chaisinanunkul N, Campbell BCV, et al. Standardized nomenclature for modified Rankin Scale global disability outcomes: consensus recommendations from stroke therapy academic industry roundtable XI. *Stroke.* 2021;52:3054-3062.
